# Reactivity disturbance suppression method for small modular reactors based on core coolant flow control

**DOI:** 10.1038/s41598-022-19243-z

**Published:** 2022-09-02

**Authors:** Hongyun Xie, Qizhi Duan, Jialin Ping, Chao Lu, Liming Zhang, Shuqiang Li

**Affiliations:** State Key Laboratory of Nuclear Power Safety Monitoring Technology and Equipment, China Nuclear Power Engineering Co., Ltd., Shenzhen, 518172 China

**Keywords:** Nuclear fusion and fission, Energy infrastructure

## Abstract

Small modular reactors (SMR) have an exceptionally wide range of applications due to their flexibility. But the reactivity of SMR is more susceptible to disturbance than that of large commercial reactors, which may cause the core power to deviate from the set value, and the limited internal space makes it difficult for SMR to compensate or adjust for reactivity disturbance by setting a sufficient number of control rods as in large commercial reactors. Therefore, in order to improve the operational stability of SMR, a method is proposed to indirectly change the nuclear fuel temperature by adjusting the coolant flow rate and thus compensate the reactivity disturbance by the Doppler effect of nuclear fuel resonance absorption. Simulation experiments show that the method can effectively eliminate reactive disturbances that cannot be completely eliminated by control rods under the conditions of restricted SMR space and limited number of control rod sets, thus providing operational stability of SMR.

## Introduction

The core power control system is an important system to ensure the safe and stable operation of the reactor^1,2^. Core power control systems are usually required to achieve two key functions, core power control and reactivity disturbance suppression^3^. In recent years, with the popularity of fully digital instrumentation and control systems in large commercial reactors, many advanced control methods based on digital instrumentation and control systems have been proposed, such as model-referenced adaptive control^4,5^, model predictive control^6–8^, fuzzy logic control^9^, linear quadratic Gaussian with loop transfer recovery^10,11^, sliding mode control^12–14^, and fractional-order control^15,16^, etc. These advanced control methods have been gradually tested and demonstrated in some Nuclear Power Plants (NPPs) with relatively high technology maturity, as the technology maturity has increased to ensure reactor safety.

**Figure 1:**
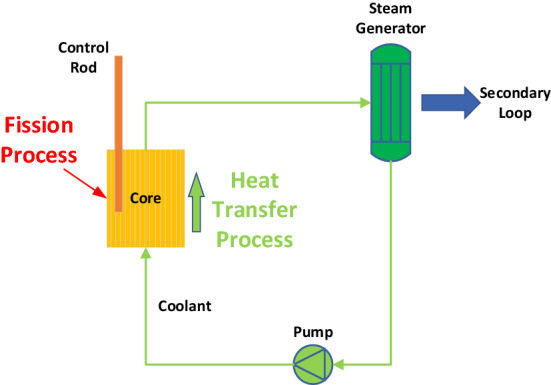
Physical processes related to the power control of nuclear reactors.

In general, the following major physical processes are associated with power control in nuclear reactors (Fig. 1): (1) heat generation in the core through chain fission reactions, (2) heat transfer processes that export fission heat to the core through thermal convection, and (3) Doppler effect caused by coolant temperature changes.

The above physical processes are mutually coupled through convective heat transfer and reactive negative temperature feedback effects.

In the power control system of a nuclear reactor, the reactivity of the reactor is usually changed by adjusting the position of the control rods in the core. This is due to the fact that the control rods contain boron elements capable of absorbing neutrons, so the length of insertion into the core determines the total number of neutrons it absorbs in the core. In addition, almost all large pressurized water reactors are equipped with a boron concentration regulation system that allows the injection of a boric acid solution into the coolant, which further improves the safety of the reactor power control.

Since large commercial pressurized water reactors are usually equipped with the above-mentioned complete reactor power control and regulation systems, and have a wealth of operational experience and data, researchers at many institutions have come up with many excellent advanced algorithms for large pressurized water reactors, which not only can respond quickly to various reactivity disturbances, but also can compensate or regulate the reactivity disturbances using the reactivity regulation system.

However, due to the limited space, small modular reactors (SMR), especially small fast modular reactors, can hardly be equipped with sufficient control rod sets as commercial nuclear reactors with large size, so the reactivity disturbances that may not be completely eliminated by control rods alone under some operating conditions with more intense reactivity disturbances. Therefore, it is necessary to study a reactivity disturbance suppression method that does not rely on control rods to compensate for the power deviation caused by reactivity disturbances according to the actual needs of modular reactors. On the other hand, the reactor models involving multiple state variables will also greatly increase the complexity of system stability analysis and dynamic performance analysis. So that, it is necessary to propose an analysis method that is convenient for engineering realization of the suppression of reactor power deviation.

Considering that SMR has more compact coolant system and smaller thermal inertia of the coolant, and the coolant flow rate of the main loop can be changed rapidly by adjusting the main pump speed, a reactivity compensation method without control rods movement or boron concentration adjustment was developed in this work. This method changes the coolant flow rate to affect its temperature when reactive disturbances occur, thereby compensating for fluctuations of reactivity using the Doppler effect of nuclear fuel resonance absorption. The analysis shows that the reactivity compensation method can effectively suppress the power deviation caused by the reactive disturbance without the participation of the control rod, and ensure the stable operation of the nuclear reactor. This work verifies the feasibility of using the temperature feedback Doppler effect of the coolant to eliminate reactivity disturbances in SMR through a series of simulation experiments, and provides a new idea of the design of a new SMR operation control strategy.

The rest of the paper is organized as follows. First, a generic nuclear power plant dynamic model containing several state variables such as neutron dynamics and thermodynamics is developed; then a formulation of the control problem on uncertain reactive perturbations is presented; after that, a flow control-based perturbation suppression scheme is designed on the basis of zero-pole analysis. Finally, the effectiveness of the reactive disturbance suppression method is verified.

## Reference design

In this paper, we use the SNCLFR-100 small modular reactor as a reference case to build a simulation model and verify the effectiveness of using the temperature feedback Doppler effect of coolant to eliminate reactivity disturbances in SMR. The main parameters of SNCLFR-100 are listed in Table 1^17^.

**Table 1:** Major parameters of SNCLFR-100.

Parameters	Value
Reactor type	SMR
Fuel type	MOX
Core power	100 $\mathrm{MW}$ (thermal)
Steam generator	Straight shell-tube type
Primary cooling model	Fully natural circulation
Core inlet temperature	400 °C
Core outlet temperature	480 °C
Design volume flow flux	$8528 \; \mathrm{ kg}/\mathrm{s}$

SNCLFR-100 is a 100 MW lead-cooled small modular reactor designed by the University of Science and Technology of China. It incorporates a number of advanced design concepts, such as monolithic arrangement and modular design, which will help simplify the system design and improve the safety performance and engineering feasibility of the reactor. The small size of SNCLFR-100 means that a small number of layout control rod sets are mainly used to regulate the power level. The SNCLFR-100's one-loop coolant is liquid metallic lead, so it is not possible to compensate for reactivity using conventional boron concentration regulation methods.

To solve the above problem, a method is proposed in this paper to indirectly compensate the reactivity perturbation by adjusting the coolant flow rate. This method uses the change of coolant flow rate to change the nuclear fuel temperature, which in turn triggers the Doppler effect of nuclear fuel resonance absorption, and the Doppler effect of nuclear fuel resonance absorption is negative feedback to the reactivity change, as the reactivity decreases when the nuclear fuel temperature increases and increases when the nuclear fuel temperature decreases. Therefore, controlling the coolant flow rate using a feedback system can indirectly compensate for the reactivity perturbation without changing the reactor size and structure.

The design of nuclear reactor control systems in engineering is usually based on a control-oriented reactor model. The control-oriented reactor model is usually a linear state space or a transfer function model that provides explicit input–output relationships. Therefore, a reasonable simplification of the structural model, the thermal hydraulic model and the nuclear reactor dynamics model of the SNCLFR-100 is required.

**Figure 2:**
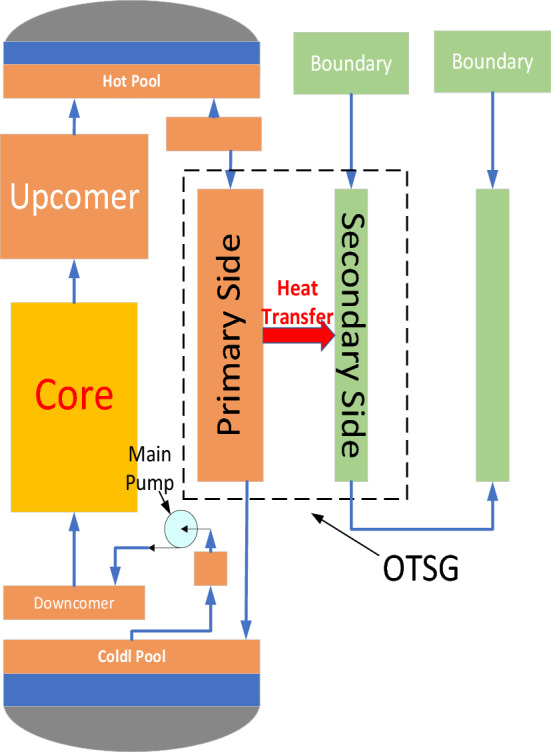
SNCPWR-100 simulation model.

The simplified SNCLFR-100 structure is shown in Fig. 2. In the primary cooling system, liquid metal lead is heated by the core as the primary coolant, and the density of the heated liquid metal lead decreases, so it flows upward into the hot pool. Meanwhile, the liquid metal lead in the primary side of the One-Through Steam Generators (OTSGs). is cooled due to the transfer of heat to the water in the secondary side, and therefore flows downward into the cold pool. Although the above natural circulation process enables the nuclear reactor to operate normally, the main pump is set up between the core and the OTSGs in order to quickly control the flow of coolant in the primary circuit.

## Control-oriented modeling

The modeling process of the control-oriented neutron dynamics model and the thermodynamic hydrodynamic model is outlined below. The detailed derivation of these equations can be found in the work of Liming Zhang et al.^3^.

### Neutron kinetic

A nonlinear core model based on the lumped parameter method was developed by using point dynamics equations for six sets of delayed neutrons and two reactive feedback mechanisms^3^, which can be given as:

1 $$\frac{\mathrm{d}n\left(t\right)}{dt}=\frac{\delta \left(t\right)-\beta }{\Lambda }+\sum_{i=1}^{6}{\lambda }_{i}{c}_{i}\left(t\right)+q\left(t\right)$$

2 $$\frac{\mathrm{d}{c}_{i}\left(t\right)}{dt}=\frac{{\beta }_{i}}{\Lambda }n\left(t\right)-{\lambda }_{i}{c}_{i}\left(t\right), \quad i=\mathrm{1,2},\dots ,6$$

where, $n$ means neutron flux; $\beta$ means delayed neutron fraction; $\Lambda$ means neutron generation time; $\delta$ means the reactivity; ${\lambda }_{i}$ means decay coefficient; ${c}_{i}$ means the delayed-neutron precursor. It should be noted that to facilitate the design of the subsequent control system, we express the core model in a linear incremental form based on the first-order Taylor expansion method:

3 $$\frac{d\Delta n\left(t\right)}{dt}=\frac{{\delta }_{0}-\beta }{\Lambda }\Delta n\left(t\right)+\frac{{n}_{0}}{\Lambda }\Delta \delta \left(t\right)+\sum_{i=1}^{6}{\lambda }_{i}{\Delta c}_{i}\left(t\right)$$

4 $$\frac{d{\Delta c}_{i}\left(t\right)}{dt}=\frac{{\beta }_{i}}{\Lambda }\Delta n\left(t\right)-{\lambda }_{i}{\Delta c}_{i}\left(t\right)$$

where, the $\Delta n$ means increment of neutron flux; ${n}_{0}$ means neutron flux in steady state; $\Delta \delta$ means the increment of reactivity; ${\Delta c}_{i}$ means the increment of the delayed-neutron precursor.

The reactivity feedback in this model is expressed as a function of the average temperature of the fuel and coolant. Thus, the total reactivity is a combination of the reactivity of the control rods and the feedback described below.

5 $$\delta \left(t\right)={\delta }_{rod}+{\alpha }_{f}\left({T}_{f}-{T}_{f0}\right)+{\alpha }_{coolant}\left({T}_{c}-{T}_{c0}\right)$$

where, the $\delta$ means reactivity; ${\delta }_{rod}$ means reactivity of rod; ${\alpha }_{f}$ means reactivity feedback of fuel; ${\mathrm{T}}_{\mathrm{f}}$ means the temperature of fuel, ${T}_{f0}$ means fuel assembly temperature in steady state; ${\alpha }_{coolant}$ means reactivity feedback of coolant in reactor core; ${T}_{c}$ means the average temperature of the coolant in the reactor core; ${T}_{c0}$ means reactor core coolant temperature in steady state.

### Thermal–hydraulic

#### Core

The reactor core is assumed to be a cylindrical region. The fission energy is released in the form of heat in the fuel elements. This heat is then transferred to the main circuit coolant. This process is described below.

6 $${\rho }_{f}{V}_{f}{C}_{f}\frac{d{\Delta T}_{f}}{dt}=\Delta P-{U}_{t}{A}_{c}\left({\Delta T}_{f}-{\Delta T}_{c}\right)$$

7 $${\rho }_{c}{V}_{c}{C}_{p}\frac{d\Delta {T}_{c}}{dt}={U}_{t}{A}_{c}\left(\Delta {T}_{f}-{\Delta T}_{c}\right)-{G}_{p}{C}_{p}\left({\Delta T}_{cout}-{\Delta T}_{cin}\right)$$

where, ${\rho }_{f}$ and ${\rho }_{c}$ means fuel density and the primary coolant density in the reactor core respectively; ${V}_{f}$ means fuel volume; ${\mathrm{V}}_{\mathrm{c}}$ means coolant volume in reactor core; ${C}_{f}$ and ${C}_{p}$ mean specific heat of fuel and primary coolant respectively; ${\Delta T}_{f}$ and ${\Delta T}_{c}$ mean fuel and primary coolant temperatures respectively; $\Delta P$ means reactor power; ${U}_{t}$ means heat transfer coefficient between fuel and primary coolant; ${A}_{c}$ means fuel assemblies area; ${G}_{p}$ means mass flow rate of primary loop; ${\Delta T}_{cout}$ and ${\Delta T}_{cin}$ mean the increment of coolant temperature in the reactor core outlet and inlet respectively.

#### Steam generator

Single-phase liquid lead and two-phase water are in primary side and secondary side of OTSGs respectively. In order to give a simple description of the control-oriented modeling, one-section model is adopted to describe the main thermodynamics of OTSGs:

8 $${\rho }_{sgp}{V}_{sgp}{C}_{p}\frac{d{\Delta T}_{sgp}}{dt}={G}_{p}{C}_{p}\left({\Delta T}_{sgpin}-\Delta {T}_{sgpout}\right)-{U}_{sg}{A}_{sg} {G}_{s}$$

9 $${\rho }_{sgs}{V}_{sgs}{C}_{s}\frac{d{\Delta T}_{sgs}}{dt}={G}_{s}{C}_{s}\left({\Delta T}_{fw}-\Delta {T}_{sgsout}\right)+{U}_{sg}{A}_{sg} {\Delta T}_{sg\Delta }$$

where, ${\rho }_{sgp}$ and ${\rho }_{sgs}$ mean coolant density in the primary and secondary side of the OTSG respectively; ${V}_{sgp}$ and ${V}_{sgs}$ mean primary and secondary side volume of OTSGs respectively; ${C}_{p}$ and ${C}_{s}$ mean specific heat in the primary and secondary side of the OTSG respectively; ${\Delta T}_{sgp}$ and ${\Delta T}_{sgs}$ mean increment of coolant temperature in the primary and secondary side of the OTSG respectively; and the ${\Delta T}_{sg\Delta }$ can be given by ${T}_{sg\Delta }={T}_{sgp}-{T}_{sgs}$; ${G}_{p}$ and ${G}_{s}$ mean mass flow rate of primary and secondary loop respectively; ${\Delta T}_{sgpin}$ and $\Delta {T}_{sgpout}$ mean inlet and outlet coolant temperatures in the primary side of OTSGs; ${U}_{sg}$ and ${A}_{sg}$ mean the heat transfer coefficient and area between the two sides of OTSGs respectively; ${T}_{fw}$ means feed-water temperature; ${T}_{sgsout}$ means outlet temperature in secondary side of OTSGs.

### Coupling process

In the SMR model, $\left(\Delta n,\Delta P\right)$, $\left(\Delta {T}_{cout},{\Delta T}_{sgpin}\right)$, and $\left({\Delta T}_{sgpout},{\Delta T}_{cin}\right)$ are three pairs of couple variables:

10 $$\Delta P=\frac{{\Sigma }_{f}V}{3.125\times {10}^{10}}\Delta n$$

11 $${\Delta T}_{sgpin}+{\tau }_{1}\frac{d{\Delta T}_{sgpin}}{dt}=\Delta {T}_{cout}$$

12 $${\Delta T}_{cin}+{\tau }_{2}\frac{d{\Delta T}_{cin}}{dt}=\Delta {T}_{sgpout}$$

where, $\Delta P$ means increment of reactor power; $\Delta n$ means increment of neutron flux; $V$ means the volume of the reactor core; ${\tau }_{1}$ and ${\tau }_{2}$ mean time delay coefficient of the heat transfer process in the SG and reactor core.

**Figure 3:**
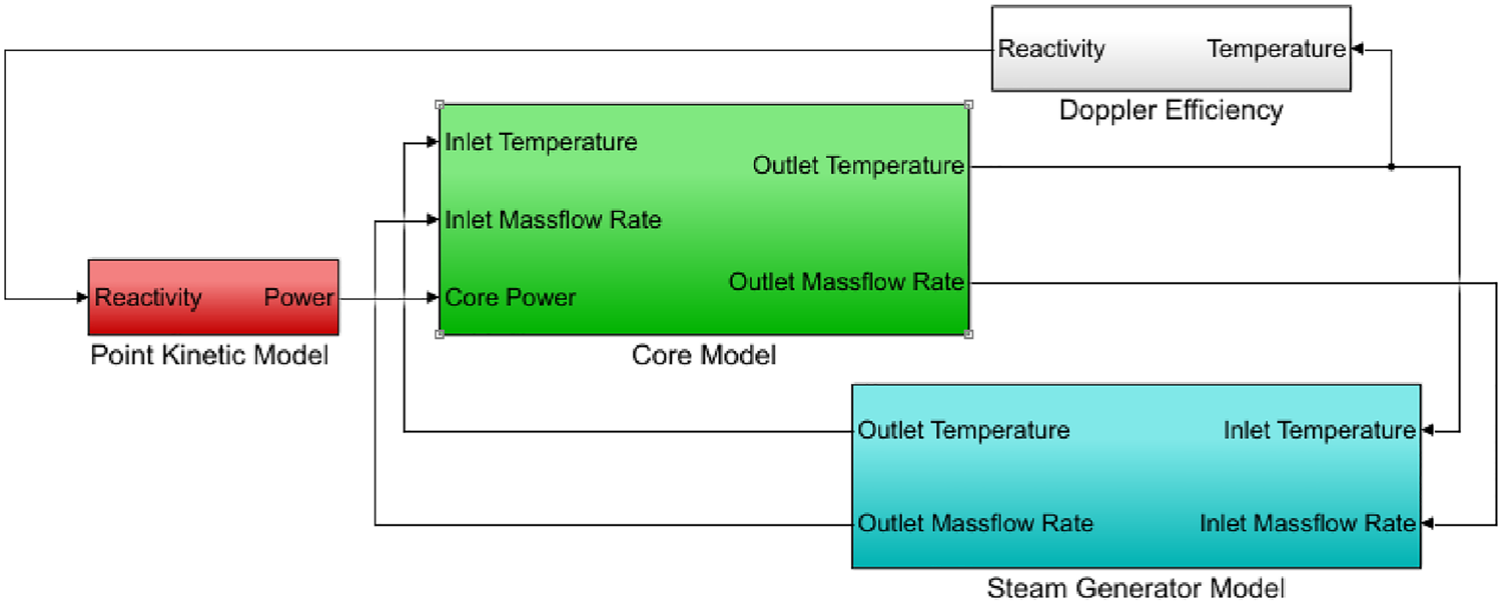
Open-loop model of reactor neutron dynamics coupled with thermal hydraulics.

MATLAB/Simulink software is used to simulate the above model. The structure of MATLAB/Simulink model can be given as Fig. 3.

**Figure 4:**
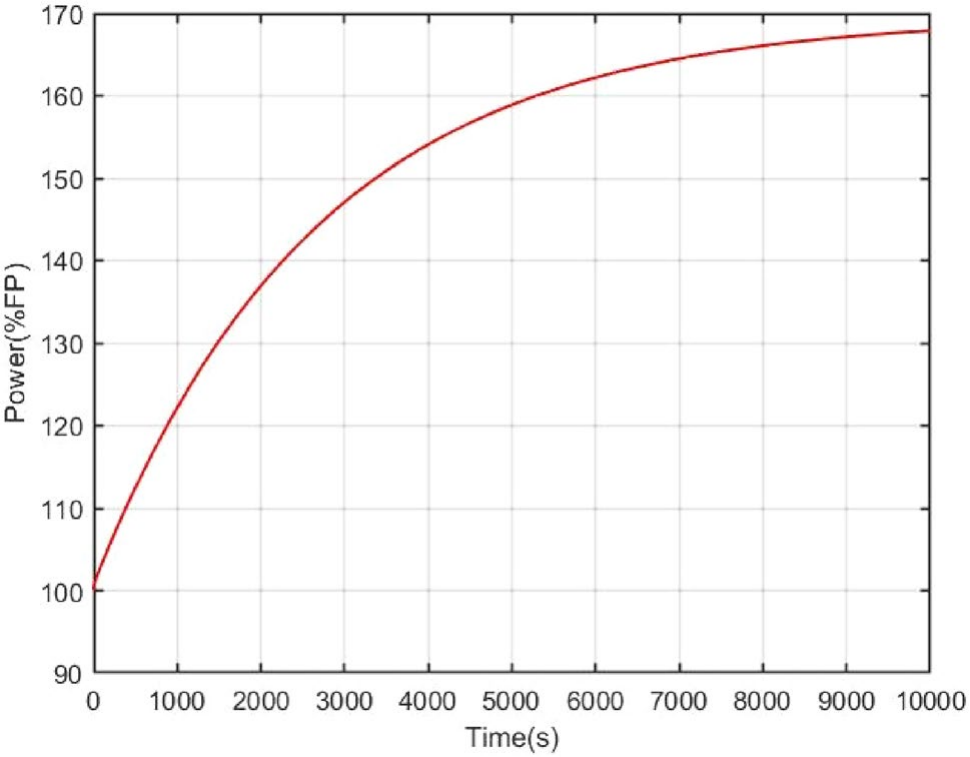
Power open-loop response under reactivity perturbation.

**Figure 5:**
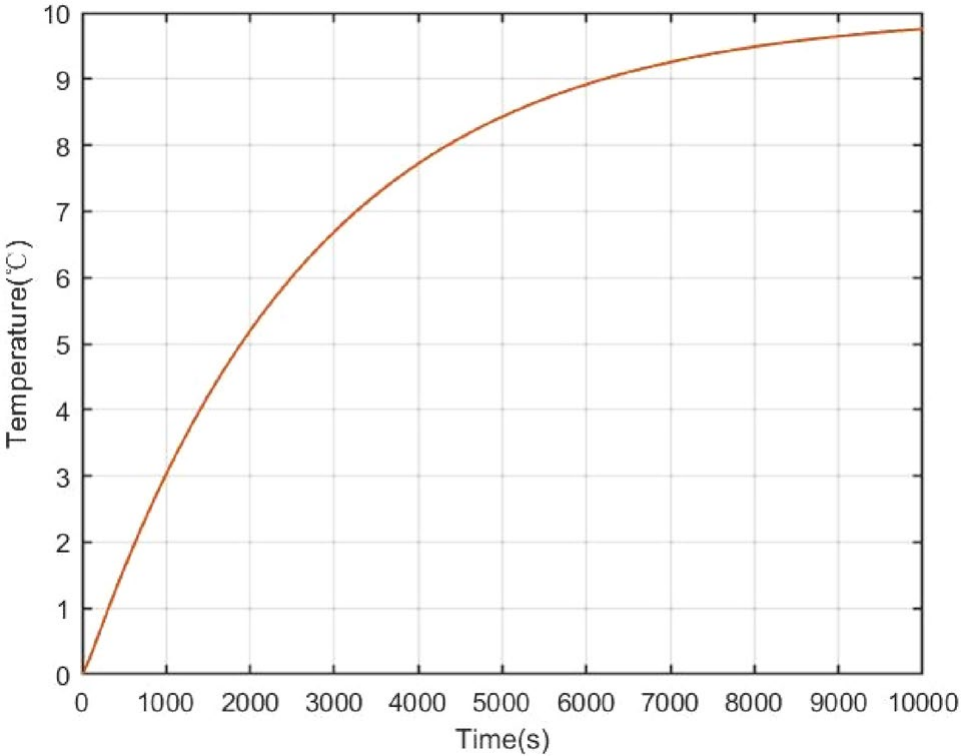
Core coolant temperature open-loop response under reactive perturbation.

**Figure 6:**
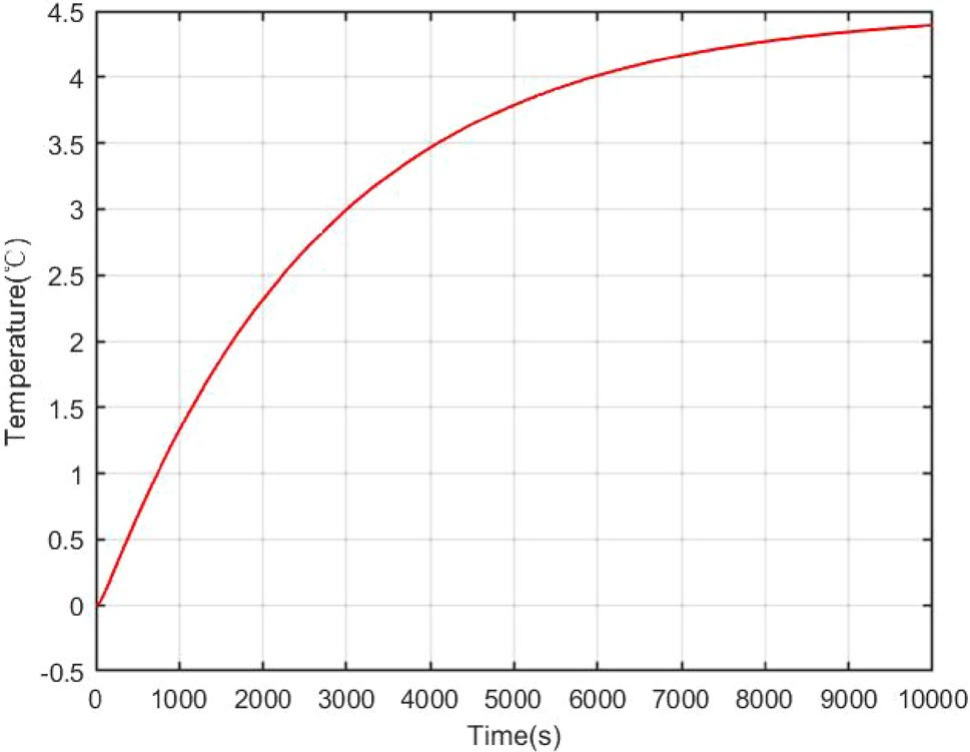
Open loop response of steam generator outlet temperature under reactive perturbation.

The open loop simulation results can be shown as Figs. 4, 5, 6. Once a reactivity disturbance occurs (as an example, the reactivity disturbance $\delta =1\times {10}^{-4}$ is introduced), the reactor power will change, although the reactor has good stability (70% change in power). Moreover, it also takes a long time for the nuclear reactor to re-stabilize to the new steady state (less than 3 h). Therefore, reactivity compensation is necessary for the NPP.

## Design of disturbance suppression scheme based on flow control

Due to the small number of control rod sets in modular reactors, additional reactivity compensation schemes need to be developed to suppress the effects of reactivity perturbations. Considering the significant negative temperature Doppler feedback effect in the nuclear fuel, the nuclear fuel temperature can be regulated by actively controlling the core coolant temperature and thus compensating the reactivity to maintain a constant power level.

**Figure 7:**
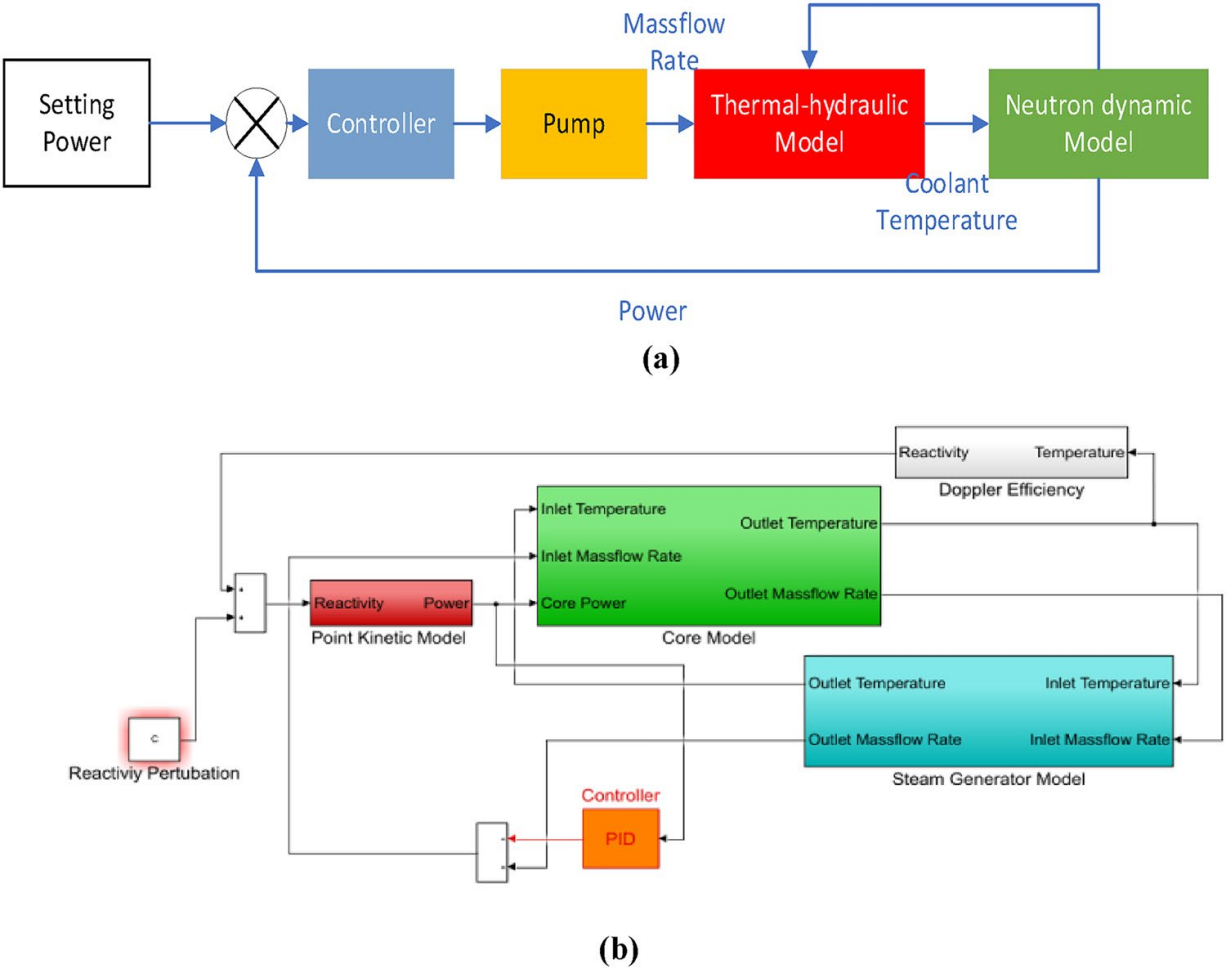
The overall of disturbance suppression: (**a**) The overall strategy; (**b**) control-oriented model.

The overall disturbance suppression strategy and the control-oriented model can be given as Fig. 7a,b respectively.

In this strategy, the reactor core power is used as feedback, and the core flow rate is changed through the regulation of the controller, so as to affect the coolant temperature in the core, and then the introduction of reactivity is changed through the Doppler effect. The controller for flow rate regulation adopts the common Proportional Integral Derivative (PID) method as follow:

13 $${{\Delta G}_{p}=K}_{p}\Delta P+{K}_{i}{\int }_{0}^{t}\Delta P\mathrm{d}t$$

where, ${\Delta G}_{p}$ means increment of mass flow rate in primary loop; ${K}_{p}$ means proportionality coefficient; ${\mathrm{K}}_{\mathrm{i}}$ means Integral coefficient.

In order to ensure that the reactor power can completely converge to the initial state, the integral coefficient ${\mathrm{K}}_{i}$ is introduced. In order to speed up the adjustment process, differential terms are not used in the controller.

To facilitate the subsequent controller design, we reformulate the model Eqs. (6–10) in a state space. Firstly, considering that $\mathrm{\Delta G}$ is the system input, Eqs. (7) and (8) can be approximately expressed as:

14 $${\rho }_{c}{V}_{c}{C}_{p}\frac{d\Delta {T}_{c}}{dt}={U}_{t}{A}_{c}\left(\Delta {T}_{f}-{\Delta T}_{c}\right)-{G}_{p0}{C}_{p}\left({\Delta T}_{cout}-{\Delta T}_{cin}\right)-\Delta {G}_{p}{C}_{p}\left({T}_{cout0}-{T}_{cin0}\right)$$

15 $${\rho }_{sgp}{V}_{sgp}{C}_{p}\frac{d{\Delta T}_{sgp}}{dt}={G}_{p0}{C}_{p}\left({\Delta T}_{sgpin}-\Delta {T}_{sgpout}\right){+\Delta G}_{p}{C}_{p}\left({T}_{sgpin0}-{T}_{sgpout0}\right) -{U}_{sg}{A}_{sg} {G}_{s}$$

where,

16 $${G}_{p}={{G}_{p0}+\Delta G}_{p}$$

${G}_{p0}$ means the flow rate of the coolant in the steady state. we can rewrite Eqs. (6), (9), (10), (15) and (16) into the state space form as follows:

17 $$\frac{\mathrm{d}{\varvec{x}}}{\mathrm{d}t}=\mathbf{A}{\varvec{x}}+\mathbf{B}{\varvec{u}}$$

where,

18 $${\varvec{x}}= {{{(\Delta n\Delta {C}_{1}\cdots\Delta {C}_{6}\Delta T}_{f}\Delta T}_{cout} {\Delta T}_{cin} {\Delta T}_{sgpin} {\Delta T}_{sgpout} {\Delta T}_{sgsout}) }^{T}$$

19 $$\mathbf{A}=\left(\begin{array}{c}\begin{array}{c}\frac{{\delta }_{0}-\beta }{\Lambda } \; \begin{array}{ccc}{\lambda }_{1}& \dots & 0 \dots 0\end{array}\\ \frac{{\beta }_{1}}{\Lambda } \; {-\lambda }_{1} \;\;0 \dots 0 \dots 0\\ \vdots \end{array}\\ \frac{{\beta }_{6}}{\Lambda } \; 0 \dots 0 {-\lambda }_{6} \; 0 \dots 0\\ \frac{{\Sigma }_{f}\Delta n{U}_{e}{\rm B}}{3.125\times {10}^{10}{\rho }_{f}{V}_{f}{C}_{f}} 0 \dots 0 -\frac{{U}_{t}{A}_{c}}{{\rho }_{f}{V}_{f}{C}_{f}} \frac{{U}_{t}{A}_{c}}{{2\rho }_{f}{V}_{f}{C}_{f}} \frac{{U}_{t}{A}_{c}}{{2\rho }_{f}{V}_{f}{C}_{f}} \; 0 \; 0 \; 0\\ 0 \dots 0 \; \frac{2{U}_{t}{A}_{c}}{{\rho }_{c}{V}_{c}{C}_{p}} -\frac{{U}_{t}{A}_{c}+2{G}_{p0}{C}_{p}}{{2\rho }_{c}{V}_{c}{C}_{p}} \frac{2{G}_{p0}{C}_{p}-{U}_{t}{A}_{c}}{{2\rho }_{c}{V}_{c}{C}_{p}}+\frac{1}{{\tau }_{2}} 0 -\frac{1}{{\tau }_{2}} 0\\ 0 \dots 0 \;\; 0 \;\; 0 -\frac{1}{{\tau }_{2}} 0 \frac{1}{{\tau }_{2}} 0\\ 0 \dots 0 \;\; 0 \;\; \frac{1}{{\tau }_{1}} 0 \frac{1}{{\tau }_{1}} \;\; 0 \;\; 0\\ 0 \dots 0 \;\; 0 -\frac{1}{{\tau }_{1}} \;\; 0 \;\; \frac{2{G}_{p0}{C}_{p}-{U}_{sg}{A}_{sg}}{{2\rho }_{sgp}{V}_{sgp}{C}_{p}}+\frac{1}{{\tau }_{1}} -\frac{{U}_{sg}{A}_{sg}+2{G}_{p0}{C}_{p}}{{2\rho }_{sgp}{V}_{sgp}{C}_{p}} \frac{{U}_{sg}{A}_{sg}}{{\rho }_{sgp}{V}_{sgp}{C}_{p}}\\ 0 \dots 0 \;\; 0\;\; 0 \;\;0 \frac{{U}_{sg}{A}_{sg}}{{2\rho }_{sgs}{V}_{sgs}{C}_{s}} \frac{{U}_{sg}{A}_{sg}}{{2\rho }_{sgs}{V}_{sgs}{C}_{s}} -\frac{{U}_{sg}{A}_{sg}+2{G}_{s}{C}_{s}}{{2\rho }_{sgs}{V}_{sgs}{C}_{s}}\end{array}\right)$$

20 $$\mathbf{B}={\left(\begin{array}{cccccccc}\frac{{n}_{0}}{\Lambda }& {0}_{1-6}& 0& 0& 0& 0& 0& 0\\ 0& {0}_{1-6}& 0& \frac{{C}_{p}\left({T}_{cout0}-{T}_{cin0}\right)}{{\rho }_{c}{V}_{c}{C}_{p}}& 0& 0& \frac{{C}_{p}\left({T}_{sgi}-{T}_{sg0}\right)}{{\rho }_{sgp}{V}_{c}{C}_{p}}& 0\end{array}\right)}^{T}$$

21 $${\varvec{u}}={\left({\delta }_{rod} \Delta {G}_{p}\right)}^{T}$$

Since the controlled variable is the core power, and considering the feedback control shown in Eq. (13), the output equation in this state space model can be expressed as:

22 $${\varvec{y}}=\mathbf{C}{\varvec{x}}$$

where,

23 $${\varvec{y}}=\Delta P$$

24 $$\mathbf{C}=\left(\begin{array}{ccc}\frac{{\Sigma }_{f}V}{3.125\times {10}^{10}}& 0& \begin{array}{cc}\cdots & 0\end{array}\end{array}\right)$$

If control rods are not used for reactivity disturbance suppression, $\mathbf{B}$ and ${\varvec{u}}$ in Eq. (17) can be expressed as:

25 $$\mathbf{B}= {\left(\mathbf{0 }\dots \mathbf{0}\;\;\frac{{C}_{p}\left({T}_{cout0}-{T}_{cin0}\right)}{{\rho }_{c}{V}_{c}{C}_{p}} 0 0 \frac{{C}_{p}\left({T}_{sgi}-{T}_{sg0}\right)}{{\rho }_{sgp}{V}_{c}{C}_{p}} 0\right)}^{T}$$

26 $${\varvec{u}}=\Delta {G}_{p}$$

According to the controller (13), the input ${\varvec{u}}$ can be rewritten as:

27 $${\varvec{u}}=\mathbf{K}{\left(\begin{array}{cc}\mathbf{C}{\varvec{x}}& v\end{array}\right)}^{T}$$

where, $\mathbf{K}=({K}_{p}{K}_{i})$, $v={\int }_{0}^{t}\Delta P\mathrm{d}t$. Then the state space model with controller can be given as:

28 $$\frac{{\varvec{d}}\widetilde{{\varvec{x}}}}{{\varvec{d}}t}=\widetilde{\mathbf{A}}\widetilde{{\varvec{x}}}$$

29 $$\widetilde{{\varvec{y}}}=\widetilde{{\varvec{C}}}{\varvec{x}}$$

where,

30 $$\widetilde{\mathbf{A}}=\left(\begin{array}{cc}\mathbf{A}+{K}_{p}\mathbf{B}\mathbf{C}& {K}_{i}\mathbf{B}\\ \mathbf{C}& 0\end{array}\right)$$

31 $$\widetilde{{\varvec{x}}}={({\varvec{x}}\boldsymbol{ }v)}^{{\varvec{T}}}$$

32 $$\widetilde{{\varvec{C}}}=(C 0)$$

In the process of designing the controller, a key problem is how to ensure the stability of the control system. The zero-pole distribution based on the transfer function is an effective feature that can reflect the stability of the system. According to Eq. (28), the poles of the system that are directly related to stability are the roots of the Eq. (33):

33 $${\varvec{d}}{\varvec{e}}{\varvec{t}}({\varvec{s}}{\mathbf{I}}_{13}-\widetilde{\mathbf{A}})=0$$

where, ${\mathbf{I}}_{13}$ means identity matrix with size $13\times 13$**.** Moreover, due to the continuous development of CAD technology, the zero-pole distribution of the transfer function can be solved directly by CAD tools to avoid the explicit derivation of the transfer function. In this work, the Simulink-based linear analysis tools were used to solve for the zero-pole distribution of the transfer function.

**Figure 8:**
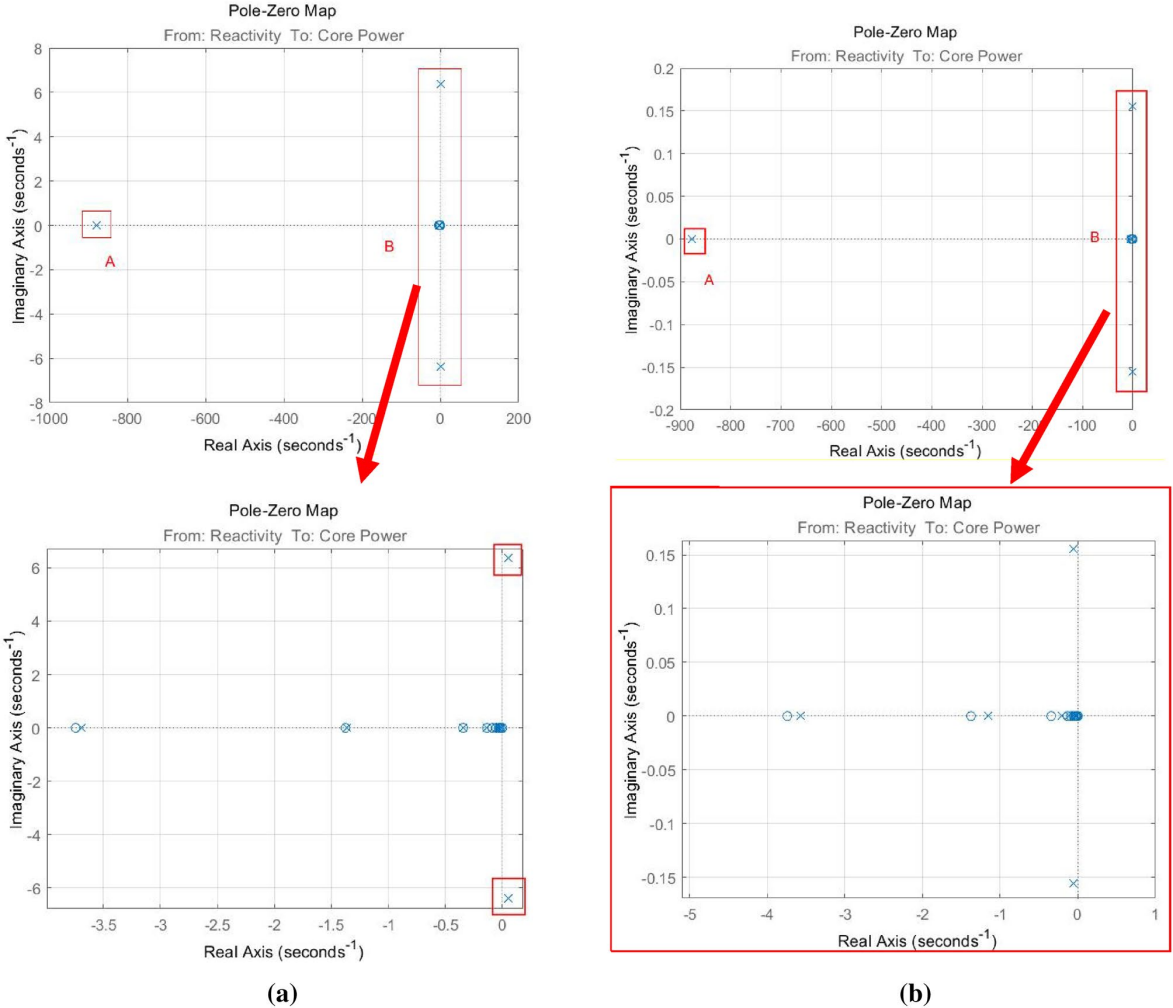
Closed loop poles and zeros distribution of the reactivity disturbance suppression system, (**a**) without correction, (**b**) with correction.

The distributions of the zero-pole distributions of closed-loop system without correction can be shown as Fig. 8a, where, the closed-loop system without correction means ${K}_{i}=1$ and ${K}_{p}=1$. Although the Proportional-Integral Module can improve the steady-state characteristics of the system and increase the response speed, it can be found that the system without correction is unstable, because there are 2 unstable poles of Eq. (32) in the right half-open plane of the complex plane (R.H.C.P) (13 poles in total). Therefore, It is necessary to find a suitable set of ${K}_{i}$ and ${K}_{p}$ to improve the stability. For the closed-loop system with correction, zeros and poles of the corrected control system can be given as Fig. 8b (where, ${K}_{i}=0.03$, and ${K}_{p}=0.2$). It is obvious that all the poles of the system are in the left half-open plane of the complex plane (L.H.C.P)^18^, which means the reactivity disturbance suppression system with PI feedback control is stable.

## Results and discussion

For verifying the effectiveness of the power compensation method presented in this work clearly, 3 sets of dynamic responses were simulated based on different power level in steady states: (a) 100%; (b) 90%; (c) 70%.

**Figure 9:**
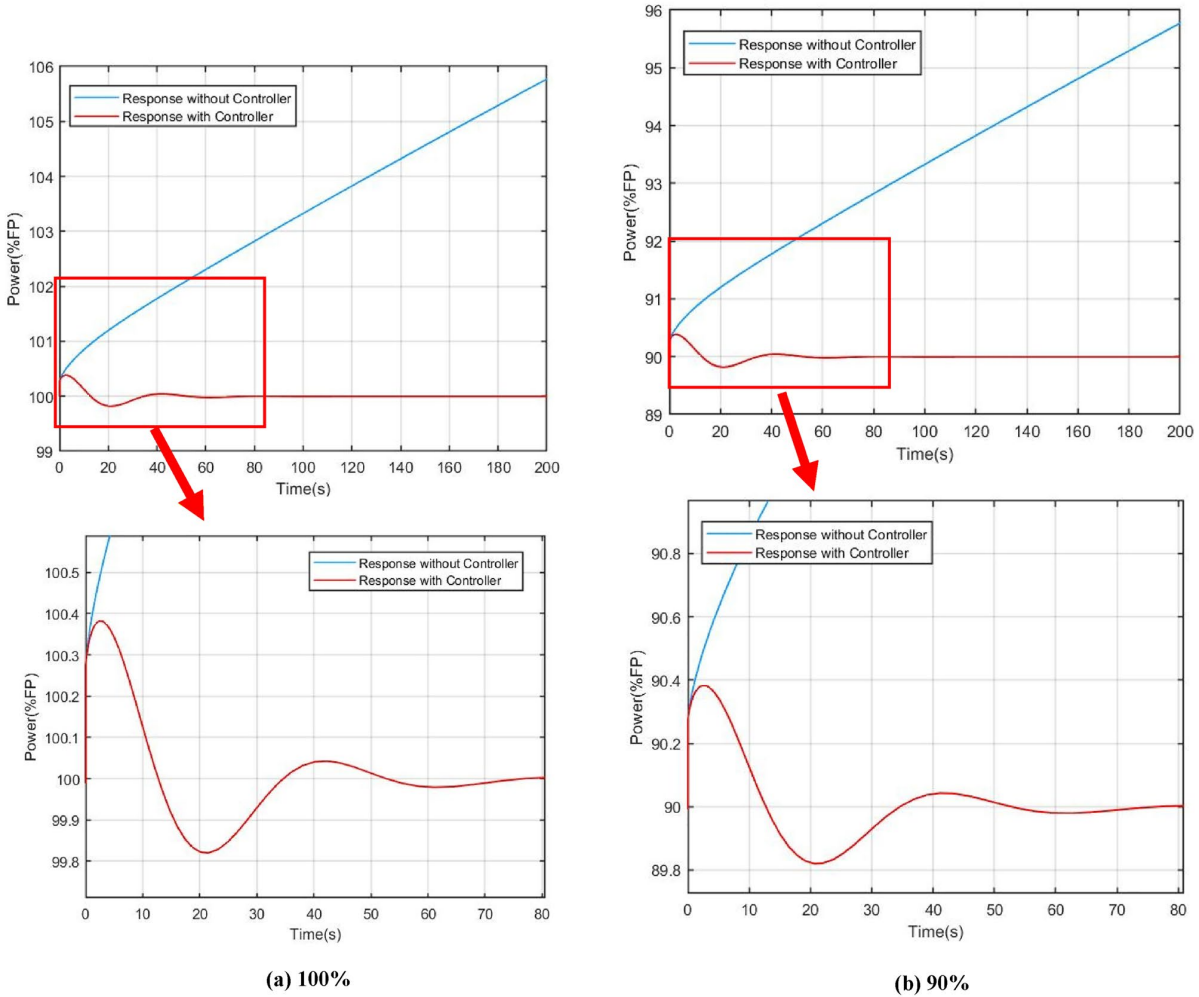

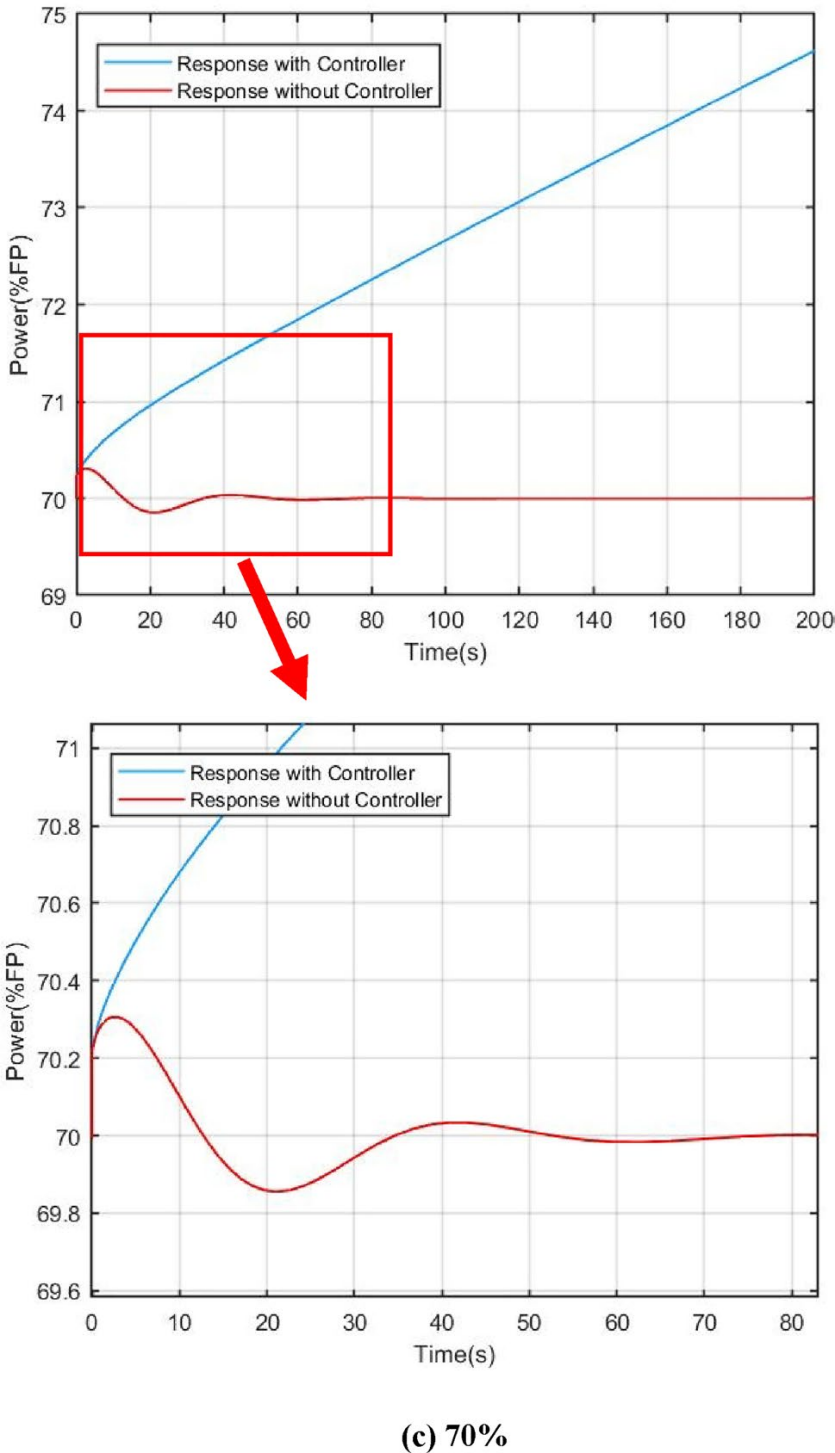
Comparison of power responses under different steady state power, (**a**) 100%, (**b**) 90%, (**c**) 70%.

**Figure 10:**
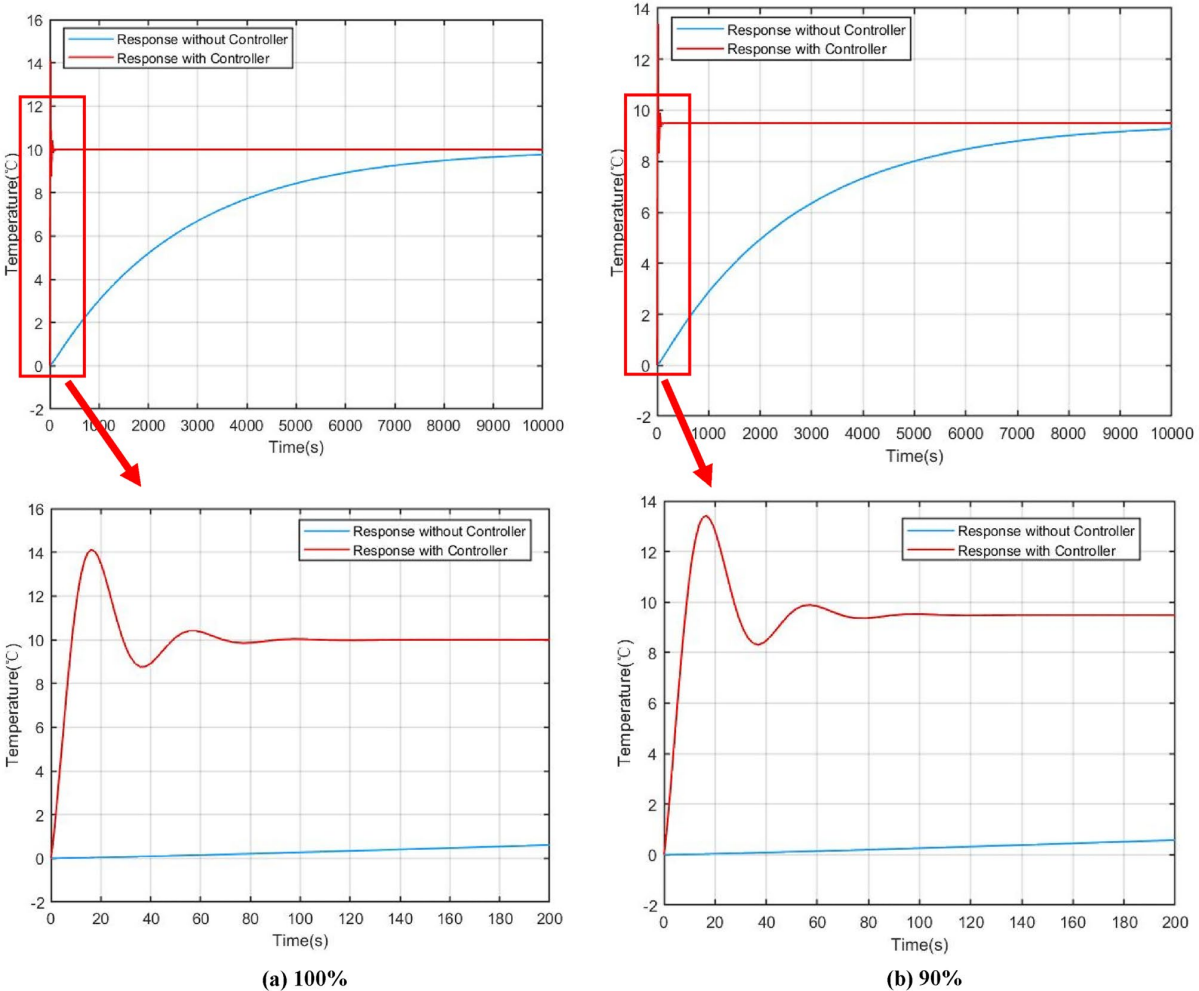

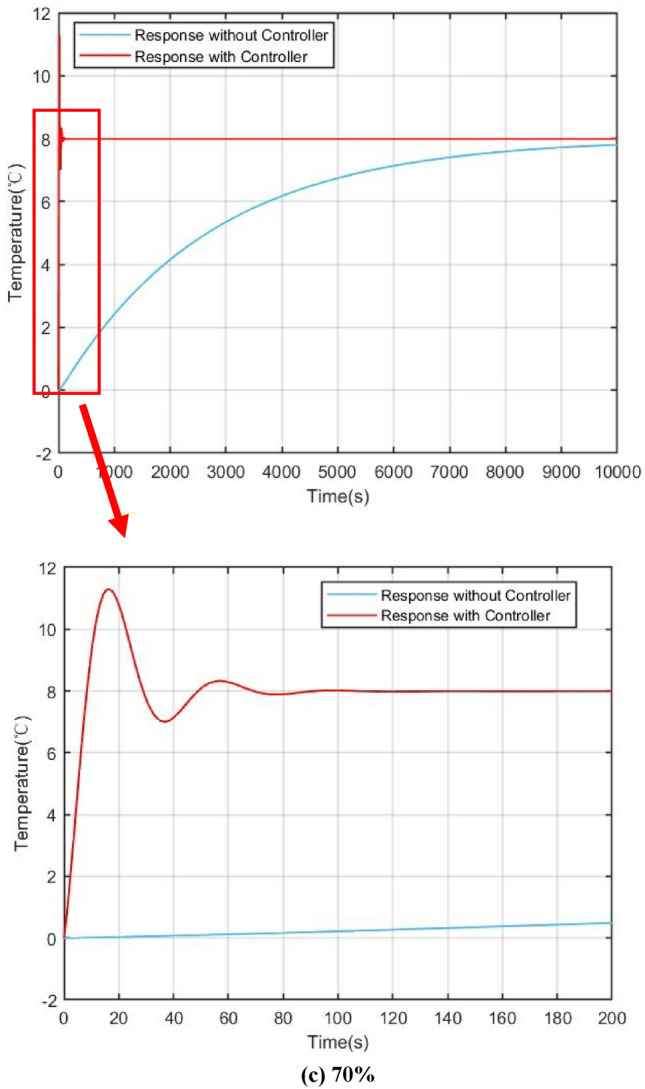
Comparison of core temperature responses with and without controller, (**a**) 100%, (**b**) 90%, (**c**) 70%.

Figures 9 and 10 show the power and core temperature dynamic response in 100%, 90% and 70% of full power (FP) respectively. When the reactivity disturbance occurs (take $\delta =1\times {10}^{-4}$ as an example), the variation of reactor power at different steady state power levels has similar variation characteristics under the influence of flow regulation: in the beginning, the power keeps rising under the action of the reactive disturbance, which causes the flow-based regulation strategy to give an abnormal regulation action, that is, to increase the core temperature by decreasing the flow. This is obviously different from the traditional flow regulation, which generally increases the flow to reduce the core temperature. The key reason for this regulation by the control strategy are that an increase in core coolant temperature provides negative reactive feedback, which suppresses reactive disturbances in the core.

Another benefit of reactive compensation is that it greatly improves the response speed of power regulation. As demonstrated by the open-loop response in the section VI, it takes several hours for the core power to return to steady state without reactivity compensation. On the other hand, it can also be found that the variation ranges and dynamic convergence time of power and core temperature are slightly different at different steady-state levels. In the simulation of the 100% full power, the adjustment time is about 120 s, while in the simulation of 70% full power, the adjustment time is reduced to 100 s. The main reason for this are that the steady-state temperature of the core varies from different steady-state power levels, which results in a change in the feedback coefficient.

In conclusion, the disturbance suppression scheme based on flow control can effectively regulate the stability of reactor power, core temperature and evaporator temperature.

## Conclusion

When modular nuclear reactors suffer from uncertain reactivity disturbance, core power may deviate from the set parameters, and the power level of reactors may be affected further. Due to the limited internal space of modular nuclear reactors, the number of control rods is small. So that it is difficult to set up control rod groups dedicated to reactive compensation for modular reactor of medium or small size.

For this purpose, a reactivity disturbance suppression method was developed. This method does not rely on control rods and boron adjustment, but compensate for the power deviation caused by reactivity disturbances according to the actual needs of modular nuclear reactors. Considering the Doppler effect of coolant and fuel temperatures, a disturbance suppression method based on coolant flow control was proposed in the work. This method changes the coolant flow rate to affect the coolant temperature when reactive disturbances occur, thereby compensating for fluctuations of reactivity. Numerical experiment showed that this method can effectively suppress the power deviation caused by reactive disturbances.

In the future, we will continue to verify the disturbance suppression method based on full scope simulator of NPPs. Moreover, the couple mode between the disturbance suppression method and power level control method also needs to be researched.

## Abbreviations

SMR: Small modular reactors
OTSGs: One-through steam generators
RHCP: Right half-open complex plane
LHCP: Left half-open complex plane
PID: Proportional integral derivative
NPP: Nuclear power plant
